# RNA modifications: importance in immune cell biology and related diseases

**DOI:** 10.1038/s41392-022-01175-9

**Published:** 2022-09-22

**Authors:** Lian Cui, Rui Ma, Jiangluyi Cai, Chunyuan Guo, Zeyu Chen, Lingling Yao, Yuanyuan Wang, Rui Fan, Xin Wang, Yuling Shi

**Affiliations:** 1 Department of Dermatology, Shanghai Skin Disease Hospital, Tongji University School of Medicine, 1278 Baode Road, Jing’an District, Shanghai, 200443 China; 2 Institute of Psoriasis, Tongji University School of Medicine, Shanghai, China; 3 Department of Respiratory and Critical Care Medicine, Shanghai Pulmonary Hospital, Tongji University School of Medicine, Shanghai, 200433 China

**Keywords:** Biochemistry, Immunology

## Abstract

RNA modifications have become hot topics recently. By influencing RNA processes, including generation, transportation, function, and metabolization, they act as critical regulators of cell biology. The immune cell abnormality in human diseases is also a research focus and progressing rapidly these years. Studies have demonstrated that RNA modifications participate in the multiple biological processes of immune cells, including development, differentiation, activation, migration, and polarization, thereby modulating the immune responses and are involved in some immune related diseases. In this review, we present existing knowledge of the biological functions and underlying mechanisms of RNA modifications, including *N*^6^-methyladenosine (m^6^A), 5-methylcytosine (m^5^C), *N*^1^-methyladenosine (m^1^A), *N*^7^-methylguanosine (m^7^G), *N*^4^-acetylcytosine (ac^4^C), pseudouridine (Ψ), uridylation, and adenosine-to-inosine (A-to-I) RNA editing, and summarize their critical roles in immune cell biology. Via regulating the biological processes of immune cells, RNA modifications can participate in the pathogenesis of immune related diseases, such as cancers, infection, inflammatory and autoimmune diseases. We further highlight the challenges and future directions based on the existing knowledge. All in all, this review will provide helpful knowledge as well as novel ideas for the researchers in this area.

## Introduction

Chemical modification occurs on many types of biological macromolecules, such as nucleic acids, sugars, lipids, and proteins, and is specific and efficient for regulating their functions.^1–4^ RNA modifications, such as *N*^6^-methyladenosine (m^6^A), 5-methylcytosine (m^5^C), *N*^1^-methyladenosine (m^1^A), *N*^7^-methylguanosine (m^7^G), *N*^4^-acetylcytosine (ac^4^C), pseudouridine (Ψ), uridylation, and adenosine-to-inosine (A-to-I) RNA editing, are RNA features that alter the canonical AUGC bases and function as emerging and critical post-transcriptional regulators.^1–3,5^ The RNA modifications catalyzed by “writer” enzymes can be removed by “eraser” enzymes.^6,7^ However, some modifications are further modified by enzymes, which we term for the first time as “modifiers”. The modifications are identified by RNA-binding proteins (RBP) known as “readers” to participate in various physiological and pathological processes.^1–3,6,7^

The immune cell abnormality in human diseases is also a research focus and progressing rapidly these years. In 2005, a study demonstrated that RNA modifications, such as m^6^A, m^5^C, m^5^U, s^2^U, or Ψ, may influence the activations of dendritic cells (DCs) and toll-like receptor (TLR)-expressing cells.^8^ Although this research was a preliminary exploration, it gave us knowledge that RNA modification could affect the biology of immune cells. In recent years, advances in new technologies and ideas have led to an increasing number of researchers focusing on the influence of RNA modifications on immune cell biology, and their roles in immune related diseases. m^6^A is the modification most frequently studied and there already have some reviews summarized its important roles in immune processes.^9,10^ Although not thoroughly and comprehensively, other RNA modifications have also been confirmed to participate in the immune cell biology and immune related diseases. However, due to the complexity of RNA modification and the diversity of immune cells, the interaction network between RNA modification and immune cells remains largely unclear, which needs to be further consummated. Hence, based on the research status, we write this review, which we hope to be helpful for researchers and promote progress in this area.

In this review, we clarify the current understanding of eight RNA modifications and focus on their critical roles in regulating immune cell biology and immune related diseases. We also highlight questions that remain to be addressed in this area and provide perspectives for further studies.

## RNA modifications

### *N*^*6*^-methyladenosine

**Fig. 1:**
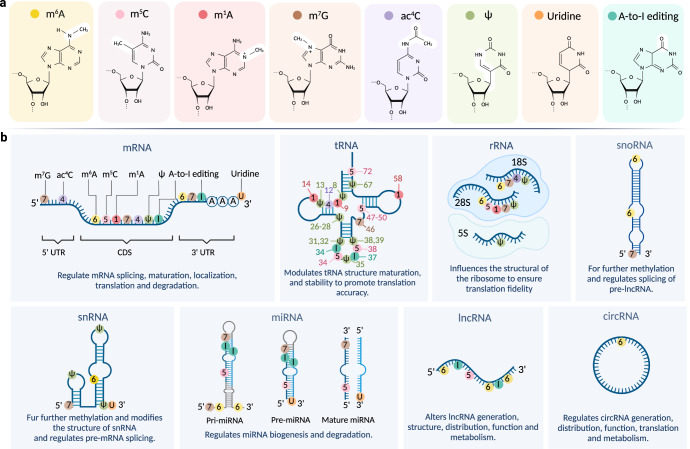
RNA modifications and their distributions on different RNA subtypes. **a** Chemical structures of eight RNA modifications. **b** Distribution of RNA modifications on different RNA subtypes. Indicated modifications are labeled at the corresponding modification sites. m^6^A *N*^6^-methyladenosine, m^5^C 5-methylcytosine, m^1^A *N*^1^-methyladenosine, m^7^G 7-methylguanosine, ac^4^C *N*^4^-acetylcytidine, ψ pseudouridine, A-to-I editing adenosine-to-inosine RNA editing, CDS coding sequence, UTR untranslated regions, pri-miRNA primary microRNA, pre-miRNA precursor microRNA

**Fig. 2:**
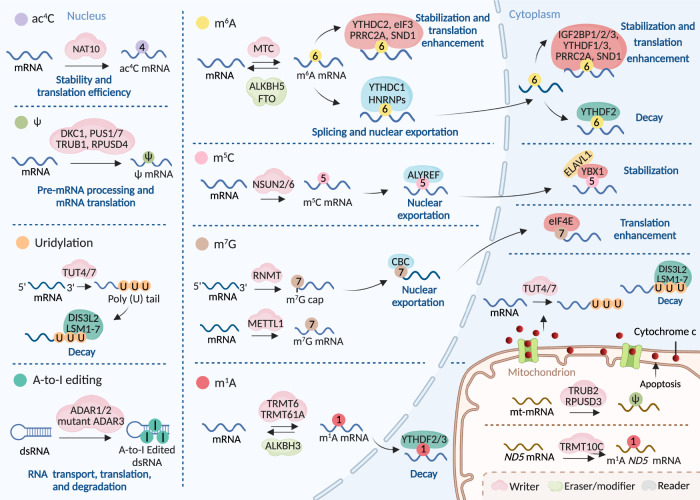
The machinery of RNA modifications and their molecular functions in mRNA. All RNA modifications included in this review can be installed on mRNA through their writers, and m^6^A as well as m^1^A modifications can be removed by indicated erasers, therefore making these RNA modifications dynamically reversible processes. Some of the RNA modifications can be recognized by their respective reader proteins, which changes the fates of target RNAs via altering generation, transportation, function and metabolization. m^6^A N^6^-methyladenosine, m^5^C 5-methylcytosine, m^1^A N^1^-methyladenosine, m^7^G 7-methylguanosine, ac^4^C N^4^-acetylcytidine, ψ pseudouridine, U Uridine, A-to-I editing adenosine-to-inosine RNA editing, dsRNA double-stranded RNA

**Fig. 3:**
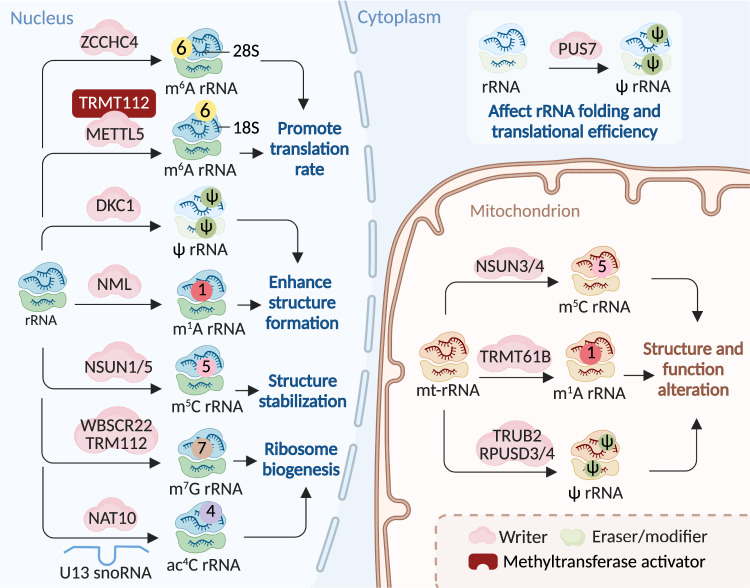
The machinery of RNA modifications and their molecular functions in rRNA. The indicated RNA modifications are installed on rRNA via their writers. These modifications occurred on rRNA alter the RNA structure, thereby regulating the function of ribosomes, which in turn affects the translation rate. The same modification can be installed by different writers in different parts of the cell. Besides, m^6^A modifications on different subunits of the ribosome can be catalyzed by different writers. Some writers also need to form a heterodimeric complex with methyltransferase activators to gain metabolic stability in cells, such as METTL5-TRMT112. m^6^A, N^6^-methyladenosine; m^5^C, 5-methylcytosine; m^1^A, N^1^-methyladenosine; m^7^G, 7-methylguanosine; ac^4^C, N^4^-acetylcytidine; ψ, pseudouridine

**Fig. 4:**
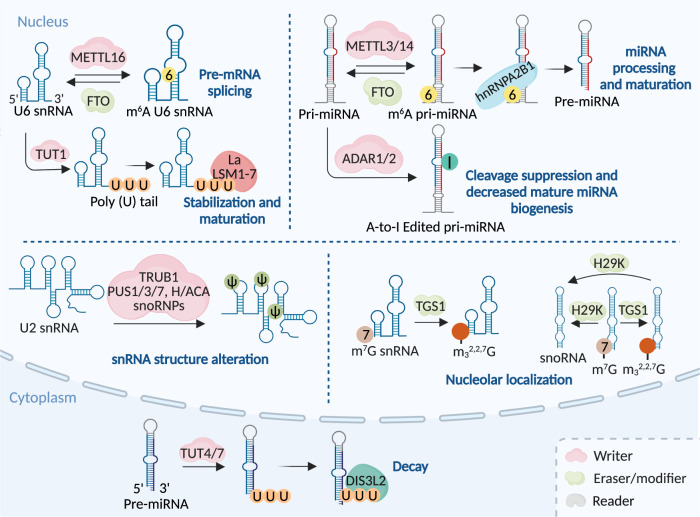
The machinery of RNA modifications and their molecular functions in snRNA, snoRNA and miRNA. The indicated RNA modifications are installed on snRNA, snoRNA and miRNA through respective writers. m^6^A modifications on snRNA and miRNA can be removed by FTO, while m^7^G modifications on snRNA and snoRNA can be removed by H29K, making RNA modifications on snRNAs, snoRNA or miRNAs dynamically reversible process. Besides, m^7^G installed snRNA and snoRNA can be further modified as m^2,2,7^G by modifier TGS1. RNA modifications affect the function of these non-coding RNAs via altering their structures, facilitating fine-tuning in various physiological processes. m^6^A N^6^-methyladenosine; m^7^G 7-methylguanosine, ψ pseudouridine, U Uridine, A-to-I adenosine-to-inosine, m_3_^2,2,7^G 2,2,7-trimethyl guanosine, pri-miRNA primary microRNA, pre-miRNA precursor microRNA

The methylation of adenosine at position N6, m^6^A modification has emerged as the most prevalent and abundant mRNA modification in eukaryotes (m^6^A/A = 0.1–0.6%).^11,12^ It appears in the full-length sequence but is enriched in the vicinity of the stop codon and the 3ʹ untranslated region (3ʹUTR) of mRNAs, within the consensus motif RRACH (R = G or A; H = A, C, or U).^13,14^ It also occurs in most non-coding RNAs, including ribosomal RNAs (rRNAs), small nuclear RNAs (snRNAs), small nucleolar RNAs (snoRNAs), microRNAs (miRNAs), long non-coding RNAs (lncRNAs), and circular RNAs (circRNAs) (Fig. 1).^15–17^

m^6^A deposition in mRNA is mostly mediated by the m^6^A methyltransferase complex (MTC) (Fig. 2 and Table 1).^18–20^ The key MTC components are methyltransferase-like 3 (METTL3), METTL14, Wilms’ tumor 1-associating protein (WTAP), vir-like m^6^A methyltransferase-associated (VIRMA, also known as KIAA1429), Cbl proto-oncogene-like 1 (HAKAI), zinc finger CCCH-type containing 13 (ZC3H13), and RNA-binding motif protein 15/15B (RBM15/15B).^18,21,22^ Among them, METTL3 is considered the only putative S-adenosylmethionine (SAM)-dependent methyltransferase with its own catalytic ability and can form a tight heterodimer with METTL14 to perform catalysis.^23–25^ The other aforementioned writers act as regulatory factors.^15,20^ Zinc finger CCCH-type containing 4 (ZCCHC4) and METTL5 mediate m^6^A formation on 28S and 18S rRNA, respectively, to accelerate the global translation rate (Fig. 3 and Table 1).^26–30^ In U6 snRNA, m^6^A is executed by METTL16 to participate in RNA splicing regulation (Fig. 4 and Table 1).^31–33^

**Table 1:** Characteristics of reviewed RNA modifications

Modifications	Target RNA	Writer	Eraser/Modifier	Reader	Biological function	Ref.
m^6^A	mRNA	METTL3/14, WTAP, VIRMA, HAIKAI, ZC3H13, METTL16, RBM15/15B	FTO, ALKBH5	YTHDF1-3, YTHDC1-2, IGF2BP1-3, HNRNPC/G/A2B1, eIF3, PRRC2A, SND1, FMR1, LRPPRC	Regulates transcription, maturation, localization, translation and degradation.	^68^
	rRNA	ZCCHC4, METTL5	N.A.	N.A.	Promotes global translation.	^26,27^
	snRNA	METTL16	FTO	N.A.	Regulates snRNA pre-mRNA splicing.	^31^
	snoRNA	METTL3/14	N.A.	N.A.	Regulates pre-lncRNA splicing.	^69^
	miRNA	METTL3/14	FTO	HNRNPA2B1	Regulates pri-miRNA processing.	^70^
	lncRNA	METTL3/14, WTAP	FTO ALKBH5	YTHDF1, YTHDF2, IGF2BP1, IGF2BP2	Regulates generation, structure, distribution, function, metabolism.	^72^
	circRNA	METTL3/14	ALKBH5, FTO	YTHDF2, YTHDF3	Regulates generation, distribution, function, translation, metabolism.	^66^
	eRNA	N.A.	N.A.	YTHDC1	Activates enhancer.	^452^
m^5^C	mRNA	NSUN2/6	N.A.	ALYREF, YBX1, FMRP	Modulates stability, export, translation and promotes mRNA-dependent repair.	^90,106,107^
	tRNA	NSUN2/3/6, DNMT2	ALKBH1, TETs	N.A.	Regulates tRNA structure and stability to ensure translation accuracy.	^78^
	rRNA	NSUN1/3/4/5	N.A.	YTHDF2	Stabilizes ribosome structural conformation to ensure translation fidelity.	^86,104^
	vtRNA	NSUN2	N.A.	N.A.	Promotes small-vault RNAs generation.	^106^
	eRNA	NSUN7	N.A.	N.A.	Protects target RNAs from degradation.	^88^
	miRNA	NSUN2	N.A.	N.A.	Affects miRNA maturation.	^453^
	lncRNA	NSUN2	N.A.	N.A.	Increases stability.	^454^
m^1^A	mRNA	TRMT6/61A/10C	ALKBH3	YTHDF1-3, YTHDC1	Regulates translation.	^115,125,126^
	tRNA	TRMT6/61A/61B/10B/10C	FTO, ALKBH1/3/7	N.A.	Stabilizes tRNA structure and promotes translational initiation.	^113,116^
	rRNA	NML, TRMT61B	N.A.	N.A.	Maintain ribosomal structure and function.	^118^
m^7^G	mRNA	METTL1, RNMT	N.A.	eIF4E, CBC	Regulates mRNA transcription elongation, slicing, export, translation and degradation.	^127,147^
	tRNA	METTL1, WDR4	N.A.	N.A.	Regulates tRNA structural integrity to promotes stability, translation ability and reduce ribosome pausing.	^133,148^
	rRNA	WBSCR22, TRM112	N.A.	N.A.	Promotes ribosome biogenesis.	^455^
	snRNA	N.A.	TGS1	N.A.	For further methylation.	^456^
	snoRNA	N.A.	TGS1, H29K	N.A.	For further methylation.	^456,457^
	miRNA	METTL1	N.A.	N.A.	Enhances miRNA processing via affecting pri-miRNA structure.	^135^
ac^4^C	mRNA	NAT10	N.A.	N.A.	Promotes mRNA stability and promote protein translation.	^158,168,169^
	tRNA	NAT10	N.A.	N.A.	Enhances its stability and indicates eukaryotic tRNA maturation.	^166,167^
	rRNA	NAT10	N.A.	N.A.	Boosts ribosome synthesis, and influences mRNA translation ability.	^160^
Ψ	mRNA	DKC1, PUS1/7, TRUB1/2, RPUSD3/4	N.A.	N.A.	Affects multiple steps in translation that could impact fidelity.	^176,178^
	tRNA	PUS1/3/7/10, TRUB1/2, RPUSD4	N.A.	N.A.	Maintains stable tRNA structure and mediate tRNA codon-anticodon base pairing to regulate translation.	^175,176,182^
	rRNA	DKC1, PUS7, TRUB2, RPUSD3/4	N.A.	N.A.	Critical for rRNA folding and controls translational fidelity.	^186^
	snRNA	PUS1/3/7, TRUB1, H/ACA snoRNPs	N.A.	N.A.	Influence structure, RNA-RNA or RNA-RBP interaction to function in pre-mRNA splicing.	^172^
Uridylation	mRNAs	TUT4, TUT7	N.A.	LSM1-7, DIS3L2, La	Promotes mRNA decay.	^202,227^
	miRNA	TUT4, TUT7	N.A.	DIS3L2	Regulates miRNA biogenesis and degradation, affects miRNAs recognizing or interacting with target sites.	^220–224,226^
	gRNAs	RET1/2	N.A.	N.A.	Initiates and promotes gRNA maturation.	^217,458^
	snRNA	TUT1	N.A.	N.A.	Promotes stabilization and maturation	^206^
	Viral RNA	TUT4, TUT7	N.A.	N.A.	Facilitates target genes degradation and involves in antiviral defense.	^230^
A-to-I editing	mRNA	ADAR1-3	N.A.	N.A.	Regulates mRNA transport, translation, and degradation and pre-mRNA splicing.	^259,268–271,275,459^
	tRNA	ADAR1-3	N.A.	N.A.	Preserves translational accuracy	^239,243–247^
	miRNA	ADAR1/2	N.A.	N.A.	Influence the biogenesis and function of miRNAs.	^241,459,460^
	lncRNA	ADAR1/2	N.A.	N.A.	Disrupts its interaction with genomic DNA or RNA.	^461,462^
	Viral RNA	ADAR1-3	N.A.	N.A.	Alters dsRNA structure, thereby suppressing innate immune responses.	^459,463^

*m*^*6*^*A* *N*^6^-methyladenosine, *m*^*5*^*C* 5-methylcytosine, *m*^*1*^*A* *N*^1^-methyladenosine, *m*^*7*^*G* 7-methylguanosine, *ac*^*4*^*C* *N*^4^-acetylcytidine, *ψ* pseudouridine, *A-to-I editing* adenosine-to-inosine RNA editing

**Fig. 5:**
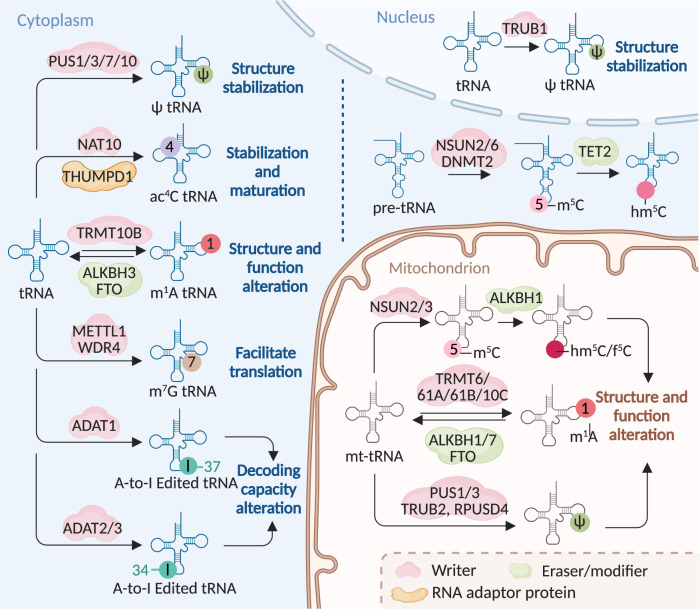
The machinery of RNA modifications and their molecular functions in tRNA. The indicated RNA modifications are installed on tRNA through indicated writers, and m^1^A modifications can be removed by ALKBH3 and FTO, while m^5^C modification on pre-tRNA can be converted into hm^5^C or f^5^C by TET2. These modifications on tRNA can alter the structure of tRNA, thereby regulating its functions to affect the translation efficiency. The same modification can be installed by different writers on tRNAs in different parts of the cell. A-to-I editing on different tRNA positions can be added by different writers. ac^4^C writer NAT10 modifies tRNAs assisted by the adaptor Tan1/THUMPD1. m^5^C 5-methylcytosine, m^1^A *N*^1^-methyladenosine, m^7^G 7-methylguanosine, ac^4^C *N*^4^-acetylcytidine, ψ pseudouridine, A-to-I adenosine-to-inosine, hm^5^C 5-hydroxymethylcytidine, f^5^C 5-formylcytidine

To date, two m^6^A erasers have been identified, both belonging to the AlkB family of the Fe (II)/α-ketoglutarate-dependent dioxygenase superfamily.^34–37^ The first eraser is fat mass and obesity-associated protein (FTO) that mediates mRNA and snRNA demethylation in m^6^A and m^6^A_m_ residues, and tRNA in m^1^A residue (Figs. 2, 4, 5 and Table 1).^35,36,38^ The other m^6^A eraser, AlkB homolog 5 (ALKBH5), only oxidatively reverses m^6^A in mRNA (Fig. 2 and Table 1).^39,40^

Many reader proteins influence the fate of m^6^A RNAs in various ways, which is largely determined by their subcellular localization. The most studied readers are the YT521-B homology (YTH) domain family members, which share the m^6^A-recognizing YTH domain but exert different effects on RNA fate.^41,42^ The YTH domain family includes YTHDF1–3 and YTHDC1–2.^41,42^ YTHDF1 and YTHDF3 actively promote protein synthesis by interacting with translation machinery, whereas YTHDF2 recruits RNA-degrading enzymes or adaptor proteins to trigger the rapid degradation of its target mRNA.^43–47^ YTHDC1 not only facilitates the decay of m^6^A-modified chromosome-associated regulatory RNAs to influence the open chromatin state and downstream transcription but also mediates mRNA splicing by recruiting and regulating pre-mRNA splicing factors.^48–50^ YTHDC2 may participate in mRNA stability and translation in an m^6^A-dependent or -independent manner.^51–53^ Insulin-like growth factor 2 mRNA-binding proteins (IGF2BPs, which include IGF2BP1–3), identify m^6^A through K homology domains to enhance mRNA stability and translation.^54,55^ The heterogeneous nuclear ribonucleoprotein (HNRNP) family members, which include HNRNPC, HNRNPG, and HNRNPA2B1, can identify m^6^A on precursor (pre)-mRNAs and/or primary (pri)-miRNAs to mediate splicing and/or nucleocytoplasmic trafficking.^56–60^ Eukaryotic initiation factor 3 (eIF3) promotes cap-independent translation upon the induction of cellular stress by recruiting the 43S complex to initiate translation.^61,62^ Both proline rich coiled-coil 2A (PRRC2A) *Staphylococcal* nuclease and tudor domain-containing 1 (SND1) function as m^6^A readers, facilitating the stabilization of modified RNA.^63,64^ In particular, m^6^A participates in transcription termination by promoting co-transcriptional R-loop formation to prevent the readthrough activity of Pol II, while it is unclear whether other readers are involved in this process.^65^ The specific roles of m^6^A in various RNA molecules are presented in Figs. 2 to 4 and Table 1.

Collectively, m^6^A modification plays a regulatory role in various cellular processes by affecting transcription, maturation, localization, function, and metabolism in different RNA classes.^14,22,66,67^ (Table 1) In mRNAs, m^6^A can affect transcription, maturation, localization, translation and degradation, eventually influencing the proteins encoded (Fig. 2).^7,68^ In rRNAs, the m^6^A1832 modification in 18s rRNA as well as the m^6^A4220 modification in 28S rRNA are required for global translation (Fig. 3).^26,27^ In snRNAs and snoRNAs, m^6^A modification may regulate snRNA pre-mRNA or pre-lncRNA splicing processes (Fig. 4).^31,69^ In miRNAs, m^6^A facilitates pri-miRNA processing by recruiting the miRNA microprocessor complex protein DGCR8 depending on HNRNPA2B1 (Fig. 4).^56,70,71^ In lncRNAs and circRNAs, m^6^A has been verified to modulate generation, structure, distribution, function and metabolism (Fig. 1), and m^6^A is also a critical translation initiator in circRNAs with coding potential.^70,72^

Although m^6^A has been widely investigated, there are still some important questions remain to be solved. For example, m^6^A is a widespread modification that can affect a variety kind of RNA, existing studies mainly focus on the effect of m^6^A on mRNA, and its effect on non-coding RNA may be of good research interest. The current study shows that the effect of m^6^A on RNA stability is bidirectional, i.e., increasing stability or promoting degradation. For this issue, it is necessary to fully consider the modification sites of m^6^A in RNAs as well as the imbalance of different readers, such as YTHDF2 and IGF2BPs. m^6^A may affect circRNA generation and circRNA-mRNA imbalance by mediating pre-mRNA splicing, which is also a potential study focus due to the recent research upsurge on circRNAs. Although existing inhibitors can interfere m^6^A levels by inhibiting writers,^25,73,74^ they are often nonspecific and affects overall m^6^A levels. It is more significant to explore gene specific m^6^A interference.

### 5-methylcytosine

m^5^C methylation occurs at position 5 of the cytidine residues of both DNA and RNA. Identified in 1958, m^5^C is described as a widespread mark in the epitranscriptome on tRNA, rRNA, mRNA, enhancer RNA (eRNA), and miRNA and is most abundant in eukaryotic tRNAs and rRNAs (Fig. 1).^75–78^

In eukaryotes, m^5^C methylation is introduced by the NOL1/NOP2/SUN domain (NSUN) family members, NSUN1–7, and DNA methyltransferase-like 2 (DNMT2) as presented in Table 1.^76,78,79^ Specific m^5^C writers catalyze different RNA subsets. According to current knowledge, cytoplasmic tRNAs are methylated by NSUN2, NSUN6, and DNMT2, while mitochondrial tRNAs are catalyzed by NSUN2 and NSUN3 (Fig. 5 and Table 1).^80–84^ rRNAs are methylated by NSUN1 and NSUN5 in the nucleus and by NSUN4 in the mitochondria (Fig. 3 and Table 1).^85–87^ mRNAs are methylated by NSUN2 and NSUN6, whereas ncRNA and eRNAs can be modified by NSUN2 and NSUN7, respectively (Fig. 2 and Table 1).^76,78,79,84,88^

Recently, some m^5^C erasers or modifiers have been concerned in RNA molecules. The known erasers/modifiers include ten-eleven translocation (TET) proteins (TET1–3) and α-ketoglutarate-dependent dioxygenase ABH1 (ALKBH1), which possess the activity of oxidizing m^5^C to 5-hydroxymethylcytidine (hm^5^C) (Fig. 5 and Table 1).^89–91^ In DNA, TETs successively convert m^5^C to hm^5^C, 5-formylcytosine (f^5^C), and 5-carboxylcytosine, the latter two of which are identified and removed by thymine DNA glycosylase;^92–95^ whereas in RNA, TETs has been only reported to convert m^5^C to hm^5^C (Fig. 5).^96–98^ ALKBH1 can successively convert m^5^C into hm^5^C and f^5^C at position 34 of cytoplasmic and mitochondrial tRNA, and the process in mitochondria is required for mitochondrial functions (Fig. 5).^91,99^ Under all circumstances, hm^5^C formation from m^5^C reduces the m^5^C modification, which is why previous studies considered TETs and ALKBH1 erasers.

Like m^6^A modification, m^5^C also involves binding proteins to change the fate of the modified RNA. The first identified mRNA m^5^C reader was RNA and export factor-binding protein 2 (ALYREF), a well-known protein complex that facilitates the nuclear export of mRNAs (Fig. 1 and Table 1).^100,101^ Y-box-binding protein 1 (YBX1) is a reader located in the cytoplasm that enhances the stability of m^5^C-modified mRNA by recruiting ELAV like RNA binding protein 1 (ELAVL1), an mRNA stability maintainer (Fig. 2 and Table 1).^102,103^ Besides, YTHDF2, also an m^6^A reader protein, could directly bind to m^5^C in RNA to modulate the distribution of m^5^C in both coding and noncoding RNA and influence rRNA maturation by regulating m^5^C levels (Table 1).^104^ Recently, Lan and colleagues presented a novel m^5^C reader, fragile X messenger ribonucleoprotein 1 (FMRP), which could be recruited to DNA damage sites by DNMT2 and promote TET1-mediated RNA m^5^C demethylation in DNA:RNA hybrids (Table 1).^90^

Generally, m^5^C plays a critical role in stabilizing both non-coding and coding RNAs. In tRNAs, m^5^C regulates RNA structure and stability and is required for translation accuracy (Fig. 5 and Table 1).^78,80,105^ m^5^C methylation at C2278 within a conserved region of 25S rRNA stabilizes the structural conformation of the ribosome, ensures translation fidelity, and recruits oxidative stress-responsive mRNA subsets to polysomes (Fig. 3 and Table 1).^86^ m^5^C methylation on vault RNAs affects their processing into derived small RNAs, while m^5^C in eRNAs protects them from degradation (Table 1).^106^ In mRNAs, m^5^C is vital for modulating stability, nuclear export, and translation (Table 1).^101,102,107–109^ For example, a subset of mRNAs with hypermethylated m^5^C sites was stabilized in an NSUN2- or YBX1-dependent manner, which influenced bladder carcinogenesis or embryonic development in zebrafish.^107^ NSUN2 enhanced the recognition of cyclin-dependent kinase inhibitor 1A (*CDKN1A*) mRNA by ALYREF, which functionally promoted the nuclear export capacity and translation of *CDKN1A* mRNA in 3T3-L1 preadipocytes.^101^

As we described above, m^5^C is abundant and required for maintaining RNA structure and stability in eukaryotic tRNAs and rRNAs, which are vital molecules in maintaining the normal physiology of almost all types of eukaryotic cells. Thus, targeting m^5^C as a therapeutic approach may have a long way to go. Fortunately, different RNAs possess different writers, and targeting specific writers can affect the function of specific RNAs. For instance, a recent study revealed that targeting NSUN3 to regulate site-specific mitochondrial RNA m^5^C modification shows therapeutic effects in combating cancer metastasis.^105^

### *N*^*1*^-methyladenosine

Identified in the 1960s, m^1^A was reported as the methylation of adenosine at position N1 and has been observed in tRNAs, rRNAs, mRNAs, and lncRNAs.^110,111^ m^1^A is inextricably linked with m^6^A modification, as not only does m^1^A rearrange to m^6^A under alkaline conditions (Dimroth rearrangement), they also share some regulators (Fig. 1).^36,112^

The current reported human m^1^A writers include nucleomethylin (NML, also known as RRP8) (for rRNA), the tRNA methyltransferase 6 non-catalytic subunit (TRMT6)–RNA methyltransferase 61A (TRMT61A) complex (for mRNA and mitochondrial tRNA), TRMT61B (for mitochondrial tRNA and rRNA), TRMT10B (for tRNA), and TRMT10C (for mitochondrial tRNA and mRNA).^113–117^ m^1^A erasers, including FTO (for tRNA) and the ALKBH family members ALKBH1 (for mitochondrial tRNA), ALKBH3 (for tRNA and mRNA), and ALKBH7 (for mitochondrial tRNA), overlap or are closely related to some m^6^A erasers. Accordingly, it has been verified that some m^6^A readers, i.e., YTH domain family proteins including YTHDF1–3 and YTHDC1, identify m^1^A modifications (Figs. 2, 3, 5 and Table 1).^36,99,118–123^

Generally, m^1^A affects RNA base pairing and subsequently influences the target RNA molecule structure and function.^115,119,124^ Human rRNAs and tRNAs contain many different m^1^A modification sites. For example, m^1^A at position 1322 of 28S rRNA promotes 60S ribosomal subunit formation and m^1^A at position 947 is essential for mitoribosomal structure and function.^113,116^ m^1^A at position 58 of tRNA is essential for tRNA structure, stability, and translational initiation; in this position, absent m^1^A may promote the generation of tRNA-derived small RNAs (tDRs), enhancing ribosome assembly and causing malignant phenotypes.^118,121^ In mRNA, m^1^A is distributed in every mRNA segment, which includes the coding sequence (CDS), 5′UTR, and 3′UTR, and its roles appear region- or subcellular location-dependent.^119,125^ Near the start codon, m^1^A might regulate translation initiation by altering the secondary/tertiary structure or reader recognition of translation initiation sites (TISs), thereby promoting translation.^125^ In mitochondria, m^1^A in the 5′UTR or CDS repressed translation, probably by affecting ribosome scanning or translation (Figs. 2, 3, 5 and Table 1).^115,126^

Because m^1^A shares some regulators, such as YTHDF1–3 and YTHDC1, with m^6^A modification, the research ideas of m^6^A can provide reference for m^1^A. Since m^1^A modification can affect RNA base pairing, we expect that it might affect the binding of miRNAs with other RNA structures, such as mRNA 3ʹUTR, lncRNA and circRNA. Competing endogenous RNAs (ceRNA) regulatory network is attracting much attention these years, and m^1^A modification may add novel conceptions to this theory.

### *N*^*7*^-methylguanosine

m^7^G refers to the RNA methylation of guanine at position N7 and is present in approximately 0.4% of all guanosine, a level similar to that of m^1^A modification.^127–129^ m^7^G is well known for the formation of the 5ʹ cap (m^7^GPPPN) structure of mature mRNA, snRNA, and snoRNA; moreover, it is enriched in all three transcript segments of mRNA 5ʹUTR, CDS, and 3ʹUTR and in pre-mRNAs.^127,130–132^ m^7^G is also present in noncoding RNAs, such as position 46 of tRNA, G1575/G1639 of 18S rRNA, and even mature and pre-miRNAs (Fig. 1).^133–135^

RNA guanine-7 methyltransferase (RNMT), METTL1–WD repeat domain 4 (WDR4) complex, and Williams–Beuren syndrome chromosomal region 22 protein (WBSCR22, also known as BUD23) are considered m^7^G writers. Activated by RNMT-activating mini-protein (RAM), RNMT is required for efficient cap methylation.^136–138^ By forming a complex with WDR4 or other partners, METTL1 has m^7^G methyltransferase activity for tRNA, internal mRNA, and pri-miRNA/miRNA.^127,135,139,140^ Requiring the methyltransferase adapter protein TRM112, WBSCR22 specifically methylates m^7^G in 18S rRNA.^141^ In most non-coding RNAs, the m^7^G cap can be lost during maturation by cleavage or further modification to m^2,2,7^G trimethylguanosine.^142^ For example, trimethylguanosine synthase 1 (TGS1) might function as a modifier, which hypermethylated the m^7^G caps of snRNAs and snoRNAs to a m^2,2,7^G cap structure, leading to their concentration in nuclear foci.^143^ The m^7^G cap can be recognized by eIF4E and the cap-binding complex (CBC) composed of CBP80 and CBP20, thereby affecting RNA maturation, nuclear export, and translation (Figs. 2 to 5 and Table 1).^144–146^

On mRNA, the m^7^G cap regulates multiple stages of mRNA processes, including pre-mRNA slicing, nuclear export, transcription elongation, translation, and degradation and indirectly augments ribosome synthesis and translation rates.^127,142,147^ On internal mRNA, m^7^G methylation might influence mRNA translation.^127^ m^7^G in tRNAs remodels the mRNA translatome by maintaining tRNA structural integrity to promote its stability, translation ability, and reduce ribosome pausing.^133,140,148,149^ However, the effects of m^7^G on rRNA have not been studied in-depth. In miRNA, m^7^G promoted miRNA processing by antagonizing G-quadruplex structures in pri-miRNA (Figs. 2 to 5 and Table 1).^135,150,151^

As we know, m^7^G is widely present in mRNAs and is a critical regulator in the translation process, therefore, it may not be a good therapic target in human diseases. The roles of m^7^G regulators may vary in different RNAs and diseases. For example, m^7^G modification on tRNA promoted the progression of lung cancer,^139^ while m^7^G modification on let-7 miRNA showed the opposite effect.^135^ m^7^G modification promoted the progression of hepatocellular carcinoma and bladder cancer,^152,153^ while it exerted an opposite opposite effect in teratoma.^154^

### *N*^*4*^-acetylcytosine

Aside from m^5^C and hm^5^C, ac^4^C (acetylation of the N4 position of cytosine) is another conserved modification in cytidine and is currently the only acetylation event described in eukaryotic RNA.^155–157^ As with many RNA modifications, ac^4^C was detected initially in tRNA and rRNA, followed by mRNA.^158,159^ In rRNA, ac^4^C is distributed in helix 34 and helix 45 near the decoding site of mammalian 18S rRNA; in tRNA, it is detected at the D-stem of tRNA^Ser/Leu^ in eukaryotes.^160–163^ In mRNA, the deposition of ac^4^C sites is detected mainly in the CDS region, and also in the 5ʹUTR (Fig. 1).^158^

*N*-acetyltransferase 10 (NAT10), an essential ATP-dependent RNA acetyltransferase, is currently considered the only writer of ac^4^C.^164^ It catalyzes ac^4^C modification in 18S rRNA, tRNA, and a broad range of mRNA.^158,160,164,165^ Two additional proteins are required in ac^4^C formation in human rRNA or tRNA, respectively. The first is the box C/D snoRNA U13, which is essential and specific for 18S rRNA acetylation by timely pre-rRNA folding.^160^ The other is THUMP domain-containing 1 (THUMPD1), a specific RNA adaptor protein harboring an RNA-binding motif that can interact with NAT10 to cooperate in tRNA acetylation (Figs. 2, 3, 5 and Table 1).^160,162^

In 18S rRNAs, ac^4^C is critical for pre-rRNA processing and ribosome synthesis and influences translation ability possibly by turning the 18S rRNA 3′ end into an environment rich in base modifications to interact with mRNA or tRNA.^160^ The function of ac^4^C formation in tRNA is not fully understood, but ac^4^C of tRNA can promote its stability and is considered a monitoring indicator of eukaryotic tRNA maturation due to the rapid tRNA degradation pathway.^166,167^ Furthermore, ac^4^C can influence mRNA translation. The presence of ac^4^C on mRNA CDS region robustly boosts mRNA stability and promotes protein translation, probably by affecting its interaction with cognate tRNAs during translation.^158,168,169^ However, ac^4^C modification on 5ʹUTR mainly affects translation initiation by directly and indirectly mediating exquisite locational specificity: ac^4^C modification immediately adjacent to a strong AUG start codon can repress translation, while ac^4^C modification downstream of a weak translation initiation site can facilitate translation (Figs. 2, 3, 5 and Table 1).^170^

As a newly identified RNA modification, ac^4^C remains largely unknown, particularly its regulators and molecular functions. Only one writer and no erasers or readers have been identified. The functions of ac^4^C in rRNA, tRNA, and the CDS as well as UTR regions of mRNAs have been reported, however, relevant studies are rare. More investigations are required.

### Pseudouridine

Identified nearly 70 years ago, Ψ is the C5-glycoside isomer of uridine, of which the C5 atom (instead of N1) of the heterocyclic ring is bonded to the C1′ atom of the pentose.^171–173^ Ψ is present in almost all kinds of RNAs, including coding and non-coding RNAs, and is highly conserved among species (Fig. 1).^79,171,174^

Thirteen writers for Ψ have been identified in humans, one of which is Dyskerin pseudouridine synthase 1 (DKC1), a catalytic subunit of the H/ACA snoRNP complex that catalyzes rRNA pseudouridylation, which requires an RNA guide for its catalytic activity.^175–177^ The remaining 12 writers are RNA-independent single pseudouridine synthases (PUSs): PUS1, PUSL1, PUS3, TRUB1, TRUB2, PUS7, PUS7L, RPUSD1–4, and PUS10; these enzymes have specific cellular localizations and RNA targets.^178–181^ To date, there are no known Ψ erasers and readers. The absence of erasers may be due to the relatively inert C–C bond formed by the ribose sugar and base, leading to the pseudouridylation process being irreversible (Figs. 2 to 5 and Table 1).^175^

Previous studies have shown that Ψ plays functional roles in RNA biogenesis, structure, stability, and function to participate in regulating gene expression.^79^ tRNAs contain many pseudouridylation sites, which are critical for maintaining stable tRNA structure and mediating tRNA codon–anticodon base pairing and are thereby involved in translation processes.^175,176,182–184^ Ψ also represses aberrant protein synthesis by altering the properties of tRNA-derived fragments.^185^ Similar to that in tRNA, Ψ is also abundant and present in various rRNA regions, aiding the formation of stable structures.^186–188^ Moreover, Ψ contributes to ribosome processing and function to ensure translational fidelity in protein synthesis.^178,189^ In snRNAs, Ψ was predicted to influence structure and RNA–RNA or RNA–RBP interactions to function in pre-mRNA splicing.^172,190–192^ Ψ is also involved in regulating pre-mRNA processing, mRNA structure, stability, translational fidelity, and termination, which is another mechanism of translation control apart from tRNA and rRNA modification (Figs. 2–5 and Table 1).^176,178,193–196^

Despite being identified several decades ago, the contributions of Ψ to multiple cellular processes are just starting to be revealed. Similar to ac^4^C, new things always take time to be understood. The elucidation of erasers and readers of Ψ will be one of the key directions in the future. Specially, Ψ have already been applied to generating highly effective COVID-19 mRNA vaccines,^197^ which is the clinical application of this modification and has potential value for further research.

### Uridylation

In addition to the most widespread homomeric poly (A) tails, uridylation, which consists of the untemplated addition, appears to be the second most prevalent modification at the 3ʹ RNA termini.^198–201^ Virtually, uridylation can occur on all classes of eukaryotic RNAs including mRNAs, and noncoding RNAs including U6 spliceosomal RNA, guide RNA (gRNA), small interfering RNA (siRNA), miRNA, Piwi-interacting RNA (piRNA), rRNA, and tRNA. Uridylation also targets viral RNA tagging (Fig. 1).^198,199,202^

In different substrates, uridylation is catalyzed by different terminal uridylyltransferases (TUTases), which belong to the noncanonical terminal nucleotidyltransferases (TENTs).^203,204^ In nuclear U6 snRNA, the U6 TUTase (TUT1) specifically added or restored at least four uridines at the 3′ end.^205,206^ TUT4 and/or TUT7 belonging to the TENT3 subfamily are the predominant writers of other cellular uridylation.^205,207–210^ Uridylation erasers or modifiers have not been reported and the uridylation readers include the LSM1-7 complex (for oligouridylation), DIS3L2 (for oligouridylation and polyuridylation), and La protein (Fig. 2 and Table 1).^211–216^

Uridylation alters RNA fate from diverse aspects, including RNA maturation, function, stability, and decay. Uridylation is essential for U6 snRNA maturation and 3′ stabilization to perform splicing function and initiating gRNA maturation.^206,217–219^ The functions of uridylation in miRNAs are diverse. For example, TUTs-mediated pre-miRNA uridylation is a critical step in miRNA biogenesis, which involves repairing or removing defective pre-miRNAs, arm switching, and Dicer processing.^220–222^ Uridylation on the miRNA 3′ end can recognize noncanonical target sites; on the other hand, it may abrogate target gene repression by directly affecting miRNA 3ʹUTR interactions.^223,224^ Moreover, the 3ʹ addition of uridine promotes miRNA degradation, which also applies to other small RNAs, such as siRNAs and piRNAs.^225,226^ Many studies have demonstrated that uridylation facilitates 5ʹ-to-3ʹ or 3ʹ-to-5ʹ mRNA decay, which is mediated by the recruitment of deadenylases, decapping enzymes, and exonucleases.^202,227^ Uridylation also regulates translation efficiency via various mechanisms, for example, mRNA destabilization, and rRNA and tRNA turnover.^202,209,228,229^ Moreover, viral RNA uridylation is involved in antiviral defense.^230–232^ Uridylated ncRNAs appear overrepresented in exosomes, indicating that uridylation directs RNA sorting into exosomes (Figs. 2, 4 and Table 1).^233^

Uridylation can act on almost all classes of RNAs in eukaryotic cells, further identification of writers and their auxiliary factors in recognizing specific RNA substrates, as well as of erasers and readers that regulate the deuridylation and decide the fate of uridylated transcripts, will no doubt be key to further understanding the regulatory network. The cell-type and disease-specific patterns of uridylation are also crucial in unraveling the roles of uridylation in cellular biology. Viral RNAs are also targets of uridylation, and the contributions of uridylation in fighting viruses and controlling transposons might be interesting topics of future research considering the current epidemic situation of COVID-19.

### Adenosine-to-inosine editing

A-to-I editing, which converts adenosines to inosines by deamination in RNA molecules, is a widespread co-transcriptional and post-transcriptional modification in mammals.^234,235^ A-to-I editing occurs widely in pre-mRNAs, mRNAs, noncoding RNAs such as miRNAs, lncRNAs as well as tRNAs, and even in virus RNAs (Fig. 1).^236–240^ The most common targets of A-to-I RNA editing are dsRNA hairpin structures forming from inverted Alu repetitive elements, which are located mainly within introns and untranslated regions and fewer in coding exons.^241–243^

A-to-I editing is the direct conversion of adenosine residues to inosine residues, which is not a conventional “writing” process, so the writers of A-to-I editing are also editors. Adenosine Deaminase TRNA Specific 1 (ADAT1) is responsible for the deamination of adenosine 37 to inosine in eukaryotic tRNA,^239,243,244^ while A-to-I conversion at position 34 of certain tRNAs is catalyzed by ADAT2 and ADAT3 (Fig. 5 and Table 1).^245–247^ Other A-to-I editing events are catalyzed by adenosine deaminases acting on RNAs (ADAR) family members, which are conserved in mammals.^248–252^ ADARs share similar functional domain structures of dsRNA-binding domains (dsRBDs) and a larger catalytic adenosine deaminase domain.^253,254^ There are three ADAR members: ADAR1 and ADAR2 deaminate double-stranded (ds)RNA, whereas ADAR3 binds to dsRNA as well as single-stranded (ss)RNA .^236,237,255^ ADAR3 lacks editing activity, and it may competitively bind to dsRNAs with other ADARs to decrease the efficiency of these enzymes.^256–258^ Different from other additive chemical modifications, A-to-I editing may not be further regulated by erasers/modifiers and readers.

Generally, this specific adenosine editing can cause transcriptomic diversity and influence the functions of the target RNAs.^259–262^ Although the probability of A-to-I editing occurrence in the coding regions is relatively low,^241,263^ studies have revealed its role in impacting the protein translation and function by altering the protein codon.^264–267^ For example, in colorectal cancer, the A-to-I editing of ras homolog family member Q (RHOQ) transcripts results in the substitution of asparagine with serine at residue 136 of RHOQ protein, leading to increased RHOQ activity and cancer invasion potential.^264^ In UTRs, A-to-I RNA editing can regulate RNA processes including transport, translation, and degradation.^259,268–271^ For example, ADAR1 directly edits 3ʹ UTR of *XIAP* and *MDM2* mRNAs to promote nuclear retention of these mRNAs.^272^ A-to-I editing facilitates the recruitment of the stabilizing RNA-binding protein human antigen R (HuR) to the 3ʹ UTR of the *CTSS* mRNA, thereby enhancing the stability and translation *CTSS* mRNA.^273^ In addition, the A-to-I RNA editing in 3ʹUTR has a potential to block the interaction between miRNAs and target genes to hinder the post-transcriptional repression activity.^274^ The A-to-I RNA editing in introns usually regulates alternative splicing processes.^268,275^ For example, ADAR1 deficiency may cause alternative splicing in intron 27 of the *ABCB1* gene to produce transcripts with retained intron, resulting in nonsense-mediated mRNA decay and decreased *ABCB1* mRNA stability.^276^ The A-to-I RNA editing in miRNAs may influence the biogenesis and function of miRNAs.^277,278^ A-to-I RNA editing also represses Alu elements in introns to form dsRNA structures, leading to altered linear mRNA and circRNAs generation (Figs. 2, 4 and Table 1).^279–281^

Some reports indicate that A-to-I RNA editing in pri- or pre-miRNA may induce local structural conformation changes, leading to cleavage suppression and decreased mature miRNA biogenesis.^282–285^ Conversely, some A-to-I RNA editing may not interfere or promote miRNA biogenesis.^282,286^ Specially, ADARs may also bind directly to miRNA precursors to promote miRNA processing by acting as an RNA-binding protein, independent of adjacent A-to-I editing events.^287–289^ The A-to-I editing in mature miRNAs or siRNAs may impact their target mRNA selection and silencing efficiency.^290–297^ In lncRNAs, A-to-I editing can affect their secondary structures, stability and interactions with other molecules.^238,298,299^ For example, the A-to-I RNA editing in lncRNAs may impact lncRNA-miRNA interactions and, consequently, change their miRNA sponge function.^300^ In tRNAs, A-to-I editing is closely associated with their decoding capacity (Fig. 5 and Table 1).^245^ In virus RNAs, A-to-I RNA editing can directly target the genome or transcriptome of RNA viruses to regulate viral pathogenicity as well as host innate immune response, which we will discuss in detail below.^301–303^

Nonetheless, there are many questions that remain to be answered in this field. How the target sites of A-to-I editing are chosen by editors? Although previous studies have identified lots of A-to-I editing sites in human RNA molecules, the significance of such editing for the vast majority of RNA sites remains unclear. Due to A-to-I editing can regulate gene expression through multiple mechanisms, it may be a potential approach to assist or replace RNA interference. Except for influencing miRNA-3ʹUTR and miRNA-lncRNA interaction, A-to-I editing may also affect miRNA-circRNA interaction, which has not been validated yet. Further investigations of RNA editing may provide lessons for precise gene editing.

## Roles of RNA modification in immune cell biology

### RNA modifications and T lymphocytes

T lymphocytes originate from bone marrow progenitors, mature in the thymus, and are transported to the periphery to fulfill immune functions after activation, proliferation, and differentiation.^304,305^ The m^5^C methyltransferase NSUN2 mediates hyperhomocysteinemia-induced interleukin-17A (IL-17A) upregulation by methylating *IL17A* mRNA and enhancing its translation in T lymphocytes.^306^ A recent study discovered that the m^7^G cap methyltransferase RNMT plays critical roles in T cell activation by specifically regulating ribosome synthesis.^142^ Enzymes modulating miRNA uridylation and uridylated miRNAs are regulated during T cell activation; TUT4 is critical for maintaining miRNA uridylation in the steady state of T lymphocytes and is downregulated during T cell activation, leading to the degradation of uridylated miRNAs.^226^ A-to-I RNA editing induced by ADAT1 prevents the sensing of endogenous dsRNAs by MDA5 to participate in thymic T cell maturation, which includes negative selection.^307,308^ Specifically, m^5^C and Ψ mRNA modification may be promising in the systemic delivery of nanoparticle formulations for regulating T cell immunity and inflammation.^309^ Many studies have uncovered the key functions of RNA modifications in the biology of multiple T lymphocyte subsets, which are presented below.

#### CD4^+^ T cells

Naive CD4^+^ T cells exit the thymus as Th0 cells and differentiate into various cell subsets following different activation signals.^310,311^ The best understood effector cell subsets include T helper (Th) cells (Th1, Th2, Th9, Th17, Th22, et al.), T follicular helper (Tfh) cells, and T regulatory (T_reg_) cells.^312–314^ Up to now, there have been some studies revealed that m^6^A participates in the biology of CD4^+^ T cells, as well as several subsets.

First, m^6^A can affect the functions of CD4^+^ T cells. For example, ALKBH5 decreases m^6^A levels in *CXCL2* and *IFNG* mRNA to enhance mRNA stability and translation, thereby promoting CD4^+^ T cell responses.^315^ m^6^A can also influence CD4^+^ T cell differentiation and subset functions, which are discussed in detail below. In particular, as CD4^+^ T cells are the target cells of HIV infection, HIV infection leads to an extensive increase in m^6^A levels in both host and viral mRNAs, thereby influencing HIV replication and viral RNA nuclear export.^316^ During the latent phase of HIV-1 infection, NSUN1 binds with HIV-1 TAR RNA at the 5′ long terminal repeat and generates its m^5^C methylation, and NSUN1 binding with TAR competes with Tat–TAR interaction, leading to hampered HIV-1 transcriptional elongation and viral latency in CD4^+^ T cells.^317^

m^5^C levels and NSUN2 expression are decreased in the CD4^+^ T cells of systemic lupus erythematosus (SLE) patients, and hypermethylated m^5^C in SLE is closely associated with the immune- and inflammation-related pathways, including the immune system, cytokine signaling, and interferon (IFN) signaling.^318^ In the CD4^+^ T cells of SLE patients, ac^4^C modification in mRNAs is highly conserved and enriched in mRNA CDS regions and participates in critical immune and inflammatory signaling in SLE pathogenesis.^319^

*Th1/Th2 cells* Th1 cells are characterized by the expression of the transcription factor T-bet and IFN-γ secretion, and participate in immune responses against intracellular pathogens.^320–322^ Th2 cells are characterized by the expression of the transcription factor GAΤA3 and IL-4/5/13 and participate in immune responses against larger extracellular pathogens.^320–322^ A preliminary study using the clustering method demonstrated that m^6^A may be involved in the Th1/Th2 imbalance and the occurrence of allergic asthma.^323^

*Th17 cells* Defined by expression of the master transcription factor RORγt and the production of the lineage cytokines IL-17/IL-22, Th17 cells participate in the elimination of bacteria and fungi and in the pathogenesis of autoimmune diseases.^324–326^ In enterotoxigenic *Bacteroides fragilis*-induced intestinal inflammation and tumorigenesis, METTL14-dependent m^6^A modification promoted the splicing and generation of miR-149-3p to regulate Th17 differentiation.^327^

*Tfh cells* With Bcl6 as the lineage-defining transcription factor, Tfh cells are a specialized CD4^+^ T cell subset essential for germinal centers and B cell responses.^328–330^ METTL3/METTL14-catalyzed m^6^A modification of *ICOS* mRNA suppressed ICOS expression, resulting in impaired Tfh cell differentiation.^331^ METTL3-catalyzed m^6^A modification on the *Tcf7* mRNA 3ʹUTR enhanced the stability of *Tcf7* mRNA, ensuring TCF-1 expression in maintaining Tfh differentiation.^332^

*T*_*reg*_ *cells* Specifically expressing FoxP3 in the nucleus, T_reg_ cells play immune regulatory roles in maintaining immune cell homeostasis and preventing immunopathology.^333–335^ *Mettl14* deficiency led to the inability to maintain the differentiation of naïve T cells into induced T_reg_ cells and the *Mettl14*-deficient T_reg_ cells exhibited impaired function in suppressing naïve T cell–induced inflammation.^336^ *Mettl3*/m^6^A deficiency in T_reg_ cells increased *Socs* mRNA levels, leading to deactivation of the IL-2–STAT5 signaling which is integral in maintaining the functions and stability of T_reg_ cells.^337^

#### CD8^+^ T cells

Naive CD8^+^ T cells proliferate and differentiate into various effector and memory cell types following different activation signals. CD8^+^ T cells can persist for years and are involved in protective immunity against intracellular pathogens and tumors.^338–341^ Many studies have demonstrated that m^6^A methylation regulators are closely associated with CD8^+^ T cell infiltration in various cancers.^342–345^ Furthermore, m^6^A methylation regulators are involved in regulating CD8^+^ T cell functions. For example, Ythdf1-deficient mice exhibited an elevated antigen-specific CD8^+^ T cell antitumor response.^346^ In tumor-associated macrophages, METTL14 deficiency led to anomalous CD8^+^ T cell differentiation, driving CD8^+^ T cell dysfunction and repressing CD8^+^ T effector cell activation.^347^ Tumor-intrinsic FTO restricted the activation and effector states of CD8^+^ T cells; knockdown of FTO impaired tumor cell glycolytic activity, which restored CD8^+^ T cell function.^347^ m^1^A levels were negatively related to CD8^+^ T effector cell proliferation in colon cancer.^348^

### RNA modifications and B lymphocytes

Generally, B lymphocytes are well known for their function of producing antibodies in the adaptive immune response; they are also key modulators of the innate immune response.^349–351^ Under antigen stimulation, mature B cells are activated and differentiate into memory B cells or plasma cells, which secrete antibodies.^352–354^ Some studies have reported that m^6^A modification also participates in B cell biology. For example, METTL14 deficiency inhibits mRNA m^6^A methylation in developing B cells and blocks IL-7-induced pro-B cell proliferation and the large-pre-B to small-pre-B transition, resulting in severe B cell development stagnation in mice.^355^ The RNA exosome cofactor MPP6, m^6^A modification, and m^6^A readers play vital roles in modulating lncRNA processing, DNA recombination, and development in B cells.^356^ m^6^A methylation was significantly decreased in the plasma cells of patients with multiple myeloma, which was due to the upregulation of FTO; FTO facilitated multiple myeloma cell proliferation, migration, and invasion by targeting HSF1–HSPs in a YTHDF2-dependent manner.^357^ In addition, ADAR1 is essential for normal B lymphopoiesis in the bone marrow and peripheral maintenance.^358^

### RNA modifications and DCs

DCs are key regulators of the innate and adaptive immune responses. They integrate signals from pathogens or other damage and present processed antigens to naïve T cells to control T cell differentiation.^359–362^ Similar to other immune cell types, the expression of the m^6^A methylation regulators in diseases is also associated with DC infiltration or depletion.^363,364^ Specially, chemokine receptor 7 (CCR7) increases lnc-Dpf3 expression by reducing m^6^A modification to prevent its degradation, and lnc-Dpf3 functions in the feedback control of DC migration and inflammatory responses by coupling the epigenetic and metabolic pathways.^365^ YTHDF1 identifies m^6^A-modified mRNAs encoding lysosomal proteases and promotes the translation of these transcripts in DCs, thereby suppressing the cross-presentation of wild-type DCs.^346^ DCs exposed to m^5^C-, m^6^A-, m^5^U-, s^2^U-, or Ψ-modified RNAs express decreased cytokines and activation markers, suggesting that nucleoside modifications repress the latent capacity of RNAs to activate DCs.^8^ *Mettl3*-mediated m^6^A modification maintained DC maturation and activation by promoting the translation of key factors, including CD40, CD80, and the TLR signaling adaptor TIRAP.^365^ Recognition of mRNA m^6^A methylation by YTHDF1 promoted the translation of lysosomal proteases in DCs and suppressed cross-priming of CD8^+^ T cells, resulting in defective immune recognition and tumor immune evasion.^346^ ADAR1 is required for the differentiation, functionality, and survival of DCs and alveolar macrophages, which involves the A-to-I editing of several coding genes and lncRNAs.^366^

### RNA modifications and natural killer cells

Natural killer (NK) cells are cytotoxic lymphocytes of the innate immune system characterized by target cell killing and cytokine production, functioning in controlling viral and intracellular bacterial infections and tumors, as well as regulating other immune cells.^367–370^ m^6^A also influences the functions of NK cells. METTL3-mediated m^6^A methylation guaranteed the sufficient response of AKT and MAPK signaling to IL-15 by raising SHP-2 expression, thus exerting critical roles in maintaining NK cell homeostasis and anti-tumor immunity.^371^ YTHDF2 is increased in NK cells activated by cytokines, tumors, and cytomegalovirus infection, and is essential for maintaining NK cell homeostasis and maturation; YTHDF2 is also required for IL-15–mediated NK cell survival, proliferation, and effector functions by forming a STAT5–YTHDF2 positive feedback loop.^372^ In addition, YTHDF2 modulates NK cell proliferation and division partially via reducing *Tardbp* mRNA stability.^372^

### RNA modifications and monocytes or macrophages

Monocytes and macrophages play an essential role in the innate immune system and present phagocytic activity to exhibit antimicrobial, homeostatic, and immunoregulatory functions.^373–376^ Due to the wide application of monocyte/macrophage cell lines such as THP-1 and RAW264.7,^377,378^ there have been many studies investigating the roles of RNA modifications in monocytes or macrophages.

Some recent studies demonstrate that m^6^A modification plays critical roles in the antiviral immunity of monocytes and macrophages. For example, m^6^A-modified HIV-1 RNA escaped RIG-I-mediated RNA sensing and IFN-I-mediated innate antiviral immune responses in differentiated human monocytic cells and primary monocyte-derived macrophages.^374^ After vesicular stomatitis virus infection, METTL3 in monocytes/macrophages translocated to the cytoplasm to promote m^6^A modification of viral RNAs. Then, the m^6^A-modified viral RNAs were reshaped with decreased double-stranded RNA loads to restrain innate sensing efficacy by MDA5 or RIG-I, resulting in inactivation of the global innate immune signaling pathways.^379^ In response to viral infection, macrophages impaired ALKBH5 enzymatic activity and induced m^6^A modification-mediated inactivation of the OGDH–itaconate pathway to inhibit viral replication.^380^ In response to DNA viruses, HNRNPA2B1 promoted m^6^A modification and nucleocytoplasmic trafficking of *CGAS*, *IFI16*, and *STING* mRNAs, thereby triggering the downstream cytoplasmic TBK1–IRF3 signaling in macrophages.^381^ DDX46 recruited ALKBH5 via its DEAD helicase domain to demethylate m^6^A-modified antiviral transcripts, impeding their nuclear exportation and translation and resulting in impaired IFN production and antiviral innate responses.^382^ Under homeostatic conditions, YTHDF3 cooperated with PABP1 and eIF4G2 to enhance FOXO3 translation by binding to the translation initiation region of *FOXO3* mRNA and functioning as a negative regulator of antiviral immunity.^381^ BCG vaccine exposure can cause increased ADAR1 expression and subsequent enhanced A-to-I editing events in human macrophages to participate in trained immunity.^383^

m^6^A modification also regulates monocyte inflammation and immune activity. METTL3-mediated m^6^A modification and YTHDF2-mediated recognition promoted *PGC1A* mRNA degradation, leading to insufficient ATP production and excessive reactive oxygen species accumulation in monocyte inflammation.^384^ In the peripheral blood immune cells from patients with colorectal cancer, m^6^A modification was the most abundant in monocytes, and the m^6^A levels in the monocytes were negatively related to the monocyte immune response.^385^

m^6^A modification is involved in various aspects of macrophage biology, including polarization, differentiation, activation, inflammation, and pyroptosis.^386–389^ For example, IGF2BP2 reads the m^6^A modification on *TSC1* and *PPARG* mRNA to regulate TSC1 and PPAR-γ expression, thereby skewing M1 macrophages to M2 activation through the TSC1–mTORC1 pathway and PPAR-γ-mediated fatty acid uptake.^386^ METTL3-mediated m^6^A modification of *Irakm* mRNA accelerated its degradation, resulting in TLR signaling-mediated macrophage activation.^386^ METTL3 increased MALAT1 levels through m^6^A methylation to downregulate USP8; the reduced USP8 decreased TAK1 ubiquitination and degradation, which promoted macrophage pyroptosis and inflammation.^389^ TUT7 functioned as a regulator in TLR4-mediated inflammation in macrophages by uridylating and thereby destabilizing the mRNAs of inflammatory mediators, including *Zc3h12a*.^227^ Through targeting the miR-21 precursor, ADAR1 reduces the generation of mature miR-21, then facilitating the polarization of macrophages toward the M2 phenotype via regulating the Foxo1-IL-10 axis.^390^

### RNA modifications and granulocytes

It is well known that granulocytes are divided into three types—neutrophils, eosinophils and basophils.^391,392^ There are relatively few and superficial studies on m^6^A regulation of granulocytes. m^6^A modification on c-*Rel* and *Rela* mRNA inactivated the NF-κB pathway to suppress IL-8 secretion, thereby inhibiting neutrophil infiltration in papillary thyroid cancer progression in a METTL3- and YTHDF2-dependent manner.^393^ Other studies only found that the expression of m^6^A methylation regulators in tumors was associated with the infiltration of granulocytes, especially neutrophils.^393–395^

To summarize, for functions, RNA modifications regulate various biological processes of immune cells, including development, differentiation, activation, migration and polarization, thus modulating the immune responses. For molecular mechanism, RNA modifications target immune cell RNAs that are responsible for those biological processes and influence RNA processes including generation, transportation, function and metabolization, leading to alterant immune cell biology. However, there are many kinds of RNA modifications and their functions are complex; immune cells are also diverse, and each cell type has its own unique cellular processes. Therefore, although the mechanism presented above is a common one explicating the interaction between RNA modifications and immune cells, the roles of a certain RNA modification in a specific immune cell need to be concretely investigated. Since the relevant research is still in its infancy, more work is needed to further improve the interaction network between RNA modification and immune cells.

## Roles of RNA modifications in immune related diseases

The immune system, consists of innate and adaptive immune, functions in the host defense against harmful antigens and immune homeostasis.^396–398^ Immune cells are important constituents of the immune system, the dysregulation of which can result in immune related diseases, such as cancers, infection, inflammatory disorders, and autoimmune diseases.^399–402^ Therefore, via regulating the biological processes of immune cells, RNA modifications can participate in the pathogenesis of immune related diseases.

### Cancers

Although previous research on cancers mainly focused on the malignant phenotypes of the cancer cell itself, in recent years, there are more and more studies on anti-tumor immunity, such as immune checkpoint, immune cell infiltration, cancer immune escape, and cancer immunotherapy.^403–407^ RNA modifications have been widely investigated in cancers, and they play vital roles in various cellular biology aspects of cancer cells, such as proliferation, metastasis, metabolism, apoptosis, and treatment resistance.^1,15,18,79,175^ Relatively, the roles of RNA modifications mediating immune cell biology in tumor immunization are not extensively and profoundly considered.

**Fig. 6:**
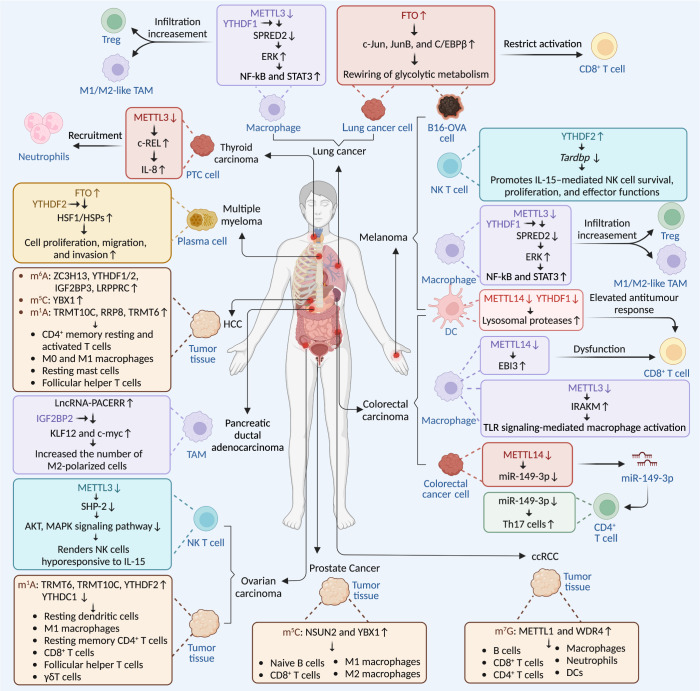
RNA modifications and immune cells in diverse cancers. RNA modifications, especially m^6^A modification, mainly play a positive role in regulating immune function in various cancers as illustrated in the figure. In melanoma and lung cancer, FTO-mediated m^6^A demethylation in tumor cells elevates the transcription factors c-Jun, JunB, and C/EBPβ, allowing the rewiring of glycolytic metabolism, thereby restricting the function of CD8^+^ T cells and inhibiting tumor growth. Besides, ablation of Mettl3 in myeloid cells promotes tumor growth and metastasis via impairing the YTHDF1-mediated translation of *SPRED2*, which enhances the activation of NF-kB and STAT3 through the ERK pathway, thereby increasing M1/M2-like tumor-associated macrophage and regulatory T cell infiltration into tumors. In melanoma, upregulation of YTHDF2 in NK cells promotes NK cell effector function and is required for IL-15–mediated NK cell survival and proliferation by targeting *Tardbp*. In melanoma and colorectal carcinoma, loss of YTHDF1 in classical DCs enhanced the cross-presentation of tumor antigens and the cross-priming of CD8^+^ T cells via increasing the m^6^A sites on transcripts encoding lysosomal proteases recognized by YTHDF1, which could be written by METTL14. In colorectal carcinoma, Mettl3- or Mettl14-deficient macrophages showed faster tumor growth via slowing down the degradation of *Irakm*, encoding a negative regulator of TLR4 signaling, or driving CD8^+^ T cells to dysfunctional ones by directly targeting *Ebi3*, respectively. Moreover, elevation of METTL14 in colorectal cancer cells promotes the differentiation of CD4^+^ T cells into Th17 cells via exosomes included miR-149-3p. In Thyroid carcinoma, METTL3 reduction in PTC cells recruits tumor-associated neutrophils into tumor tissue through IL-8, thereby further promoting tumor development, while in ovarian carcinoma, depletion of METTL3 in NK cells inhibits cell infiltration ability and function, leading to accelerated tumor development via reducing SHP-2 expression as well as the activation of AKT and MAPK signaling pathway. In multiple myeloma, upregulation of the demethylase FTO in plasma cells plays a tumor-promoting and pro-metastatic role in MM by targeting HSF1 which could be recognized by YTHDF2. In pancreatic ductal adenocarcinoma, LncRNA-PACERR increased the number of M2-polarized cells and facilized cell proliferation, invasion and migration via binding to IGF2BP2 to enhance the stability of KLF12 and c-myc, thereby activating KLF12/p-AKT/c-myc pathway through binding to miR-671-3p. Extensive bioinformatics analysis revealed the potential key roles of RNA modifications other than m^6^A modification in immune cell infiltration in diverse types of tumors. DC dendric cell, NK T cell natural killer T cell, PTC papillary thyroid carcinoma, HCC hepatocellular carcinoma, ccRCC clear cell renal cell carcinoma, TAM tumor-associated macrophage, HSF1 heat shock factor 1

The most reported RNA modification mediating immune cells in cancers is their influence on immune cell infiltration of tumors.^408–410^ m^6^A modification and multiple m^6^A regulators have been verified to be closely associated with the infiltration of various immune cells in plenty of human cancers.^408,409,411^ Relatively less, m^5^C, m^1^A, m^7^G, ac^4^C, and Ψ are also found to be related to immune cell infiltration in cancers (Fig. 6).^412–417^ However, most of these studies investigated the RNA modifications and regulators in cancer tissues and cells, but not in immune cells, and they did not elaborately explain how RNA modification disorders affect immune cell infiltration. Here, we propose an idea that chemokines secreted by tumors may be an intermediate medium regulated by RNA modification in this process.

There have been some studies exploring the RNA modification dysregulations in immune cells in the tumor immune microenvironment (TIME) and their roles in cancer progression.^13,418–420^ As expected, m^6^A is the most extensive and in-depth modification inquired. METTL3 is downregulated in tumor-infiltrating NK cells, which affects *Ptpn11* m^6^A modification and downstream IL-15-induced signaling, leading to homeostasis disruption, impaired infiltration, and function of NK cells in TIME, resulting in cancer development.^371^ Through targeting and inhibiting the stability of *Tardbp* mRNA, YTHDF2 is involved in maintaining NK cell homeostasis, maturation, IL-15–mediated survival, and antitumor activity.^372^ m^6^A modification can also influence macrophage reprogramming by mediating *SPRED2* translation, and, thereby regulating the activation of NF-κB and STAT3 signaling; METTL3 deficiency impairs the YTHDF1-mediated translation of *SPRED2*, orchestrating growth, metastasis, and anti-PD-1 therapeutic efficacy of cancer.^421^ Another study confirmed this biological effect of m^6^A in macrophages from another aspect that loss of *Mettl3* impairs the TLR4 signaling in macrophage activation by reducing *Irakm* mRNA degradation.^386^ Additionally, lncRNA-PACERR induces the polarization of pro-tumor macrophages in an IGF2BP2 and m^6^A-dependent manner.^422^ YTHDF1 negatively regulates the anti-tumor immune responses of DCs by promoting the translation of m^6^A-modified mRNAs encoding lysosomal proteases to impair immune recognition, leading to tumor immune evasion.^346^ In T cells, m^6^A modification targets specific genes to control T cell differentiation and maintains the suppressive effects of Tregs, functioning as a negative regulator in the anti-tumor immune responses.^337^ CD8^+^ T cells are direct effector cells of anti-tumor immunity, but there are few studies revealing the m^6^A disorders in this cell type in cancers. Nevertheless, m^6^A can influence the anti-tumor response of CD8^+^ T cells via controlling the biological processes of other related cells in the TIME, such as tumor cells, macrophages, and DCs.^346,347,423^ Especially, the progression of multiple myeloma, a B-cell lymphoma, is mediated by m^6^A in an FTO and YTHDF2 dependent manner.^357^ Relatively, there are no comprehensive studies on the biological functions and molecular mechanisms of other modifications regulating immune cells in cancers till now. (Fig. 6 and Table 2).

**Table 2:** Main functions of RNA modifications on various immune cells

Type of Immune cell	RNA modifications	Regulatory enzymes	Target RNA	Main functions	Ref.
T cells	m^5^C	NSUN2	*IL17A*	Enhances *IL-17A* mRNA translation.	^306^
(uncategorized)	m^7^G	RNMT	*TOP*	Modulates ribosome synthesis and activate T cells.	^142^
CD4^+^ T cells	m^6^A	ALKBH5	*CXCL2*, *IFNG*	Enhances the pathogenicity of CD4^+^ T cells.	^315^
(uncategorized)		METTL3	*Cd40*, *Cd80*, *Tirap*	Promotes DC function in CD4^+^ T-cell activation.	^365^
	m^5^C	NSUN1	HIV TAR RNA	Hamper HIV-1 transcriptional elongation and viral latency in CD4^+^ T cells.	^317^
		N.A.	N.A.	Associated with immune system, cytokine signaling and interferon signaling in SLE.	^318^
	ac^4^C	N.A.	*USP18*, *GPX1*, *RGL1*	Regulates mRNA catabolic processes and translational initiation in SLE.	^319^
	Uridylation	TUTases	N.A.	Reduces the stability of miRNAs and promotes CD4^+^ T cell activation.	^226^
	A-to-I editing	ADAT1	dsRNA	Participates in thymic T cell maturation	^307,308^
Th1/Th2 cells	m^6^A	N.A.	N.A.	Influences the Th1/Th2 imbalance in allergic asthma.	^323^
Th17 cells	m^6^A	METTL14	miR-149	Regulates Th17 differentiation in intestinal inflammation and malignancy.	^327^
Tfh cells	m^6^A	METTL3, METTL14	*ICOS*	Attenuates Tfh cell differentiation.	^331^
		METTL3	*Tcf7*	Activates Tfh transcriptional program to maintain Tfh differentiation.	^332^
T_reg_ cells	m^6^A	METTL14	N.A.	Facilitates the differentiation of T_reg_ and suppress the inflammatory response in IBD.	^336^
		N.A.	*Socs*	Maintains the functions and stability of T_reg_ cells.	^337^
CD8^+^ T cells	m^6^A	N.A.	N.A.	Regulates CD8^+^ T cells infiltration in cancers.	^342–344^
		YTHDF1	mRNAs encoding lysosomal proteases	m^6^A modification in DCs suppresses the cross-priming of CD8^+^ T cells.	^346^
		METTL14, YTHDF2	*Ebi3*	m^6^A in macrophages maintains CD8^+^ T cell differentiation and activation.	^423^
		FTO	c-Jun, JunB, C/EBPβ	Restricts glycolytic metabolism of cancer cells to activate CD8^+^ T cells.	^347^
	m^1^A	N.A.	N.A.	Negatively related to CD8^+^ proliferation ability of T effector cells in colon cancer.	^348^
	m^5^C, Ψ	N.A.	N.A.	Influences immune responses of CD8^+^ T cells.	^309^
B cells	m^6^A	METTL14	N.A.	Mediates IL-7-induced cell proliferation of pro-B cell and large-pre-B-to-small-pre-B transition.	^355^
		METTL3	lncRNAs	Promotes DNA recombination and development in B cells.	^356^
		FTO, YTHDF2	*HSF1*	Suppresses proliferation, migration, and invasion in plasma cells of multiple myeloma.	^357^
	A-to-I editing	ADAR1	N.A.	Critical for normal B lymphopoiesis in the bone marrow and peripheral maintenance.	^358^
DCs	m^6^A	N.A.	N.A.	Associated with the infiltration or depletion of DCs cancers and IBD.	^363,364^
		N.A.	lnc-Dpf3	Facilitates DC migration and inflammatory responses functions in a feedback manner.	^447^
		METTL3	*Tirap, Cd40, Cd80*	Activates DCs through TLR4/NF-κB signaling pathway and T-cell activation.	^365^
		YTHDF1	mRNAs encoding lysosomal proteases	Restricts cross-priming of CD8^+^ T cells mediated by DCs.	^346^
	m^6^A/Ψ	N.A.	N.A.	May influence the activations of DCs.	^8^
	A-to-I editing	ADAR1	N.A.	Essential for the differentiation, functionality, and survival of DCs.	^366^
NK cells	m^6^A	METTL3	*Ptpn11*	Maintains homeostasis and anti-tumor immunity of NK cells.	^371^
		YTHDF2	*Tardb*	Inhibits IL-15–mediated NK cell survival, proliferation, and effector functions.	^372^
Macrophages and/or monocytes	m^6^A	METTL14, YTHDF1	*Socs1*	Declines macrophage responses to acute bacterial infection.	^387^
		YTHDF2	*MAP2K4, MAP4K4*	Promotes LPS-induced inflammatory response in macrophages.	^388^
		METTL14, YTHDF2	*Ebi3*	Regulates macrophages-mediated CD8^+^ T cell differentiation and activation to inhibit tumor growth.	^423^
		N.A.	HIV-1 RNA	Facilitates HIV-1 escaping from innate antiviral immune responses of macrophages.	^374^
		METTL3	viral RNAs	Limits the innate sensing efficacy of macrophages for viral RNA.	^379^
		ALKBH5		Inhibits viral replication in macrophage.	^380^
		hnRNPA2B1	CGAS, IFI16, STING	Facilitates immune response to DNA viruses in macrophages.	^434^
		ALKBH5	antiviral transcripts	Increases interferon production and antiviral innate responses in macrophages.	^382^
		YTHDF3	*FOXO3*	Inhibits antiviral immunity under homeostatic conditions in macrophages.	^381^
		METTL3, YTHDF2	*PGC-1α*	Increases ROS accumulation and proinflammatory cytokines level in inflammatory monocytes.	^384^
		N.A.	N.A.	Negatively related to the immune response of monocytes in colorectal cancer.	^385^
		IGF2BP2	*TSC1, PPAR-γ*	Promotes M2 macrophages differentiation.	^443^
		METTL3	*Irakm*	Activate macrophages via TLR signaling.	^386^
		METTL3	MALAT	Promotes pyroptosis and inflammation of macrophages.	^389^
		N.A.	N.A.	Possibly promotes infiltration of macrophages in colorectal cancer.	^395^
	Uridylation	TUT7	*Zc3h12a*	Stabilize *IL6* mRNA expression in TLR4-mediated inflammation in macrophages.	^227^
	A-to-I editing	ADAR1	N.A.	Promotes the differentiation, functionality, and survival of and alveolar macrophages.	^366^
		ADAR1	N.A.	Participates in trained immunity	^383^
		ADAR1	miR-21 precursor	Reduces the generation of mature miR-21, therefore facilitating the polarization of macrophages toward the M2 phenotype via Foxo1-IL-10 axis.	^390^
Granulocytes	m^6^A	METTL3	c-Rel, RelA	Inhibit neutrophil infiltration in papillary thyroid cancer progression.	^464^
		N.A.	N.A.	Related to the infiltration of neutrophils in breast cancer and colorectal cancer.	^393–395^

*m*^*6*^*A* *N*^6^-methyladenosine, *m*^*5*^*C* 5-methylcytosine, *m*^*1*^*A* *N*^1^-methyladenosine, *m*^*7*^*G* 7-methylguanosine, *ac*^*4*^*C* *N*^4^-acetylcytidine, *ψ* pseudouridine, *A-to-I editing* adenosine-to-inosine RNA editing, *DC* dendritic cell, *SLE* systemic lupus erythematosus, *IBD* inflammatory bowel disease, *ROS* reactive oxygen species, *TLR* toll-like receptors, *LPS* Lipopolysaccharide, *HIV* human immunodeficiency virus, *dsRNA* double-stranded RNA

Despite all this, the roles of RNA modifications mediating immune cell biology in cancer immunization remains largely unclear. There seem to be some paradoxes as well as enlightenments. For instance, as we discussed above, m^6^A deficiency will lead to the disability of some anti-tumor immune cells, whereas YTHDF1 deficiency enhances anti-tumor immune responses. In this regard, we think researchers should comprehensively consider the other roles of writers, erasers, and readers, not just focusing on their regulation of RNA modifications. Moreover, as we know, RNA modifications are vital modulators of normal cell biology, and it is easy to understand that their delicacy may cause the disability of immune cells; but we don’t know what will happen if these modifications are excessive, and whether there is a balance. Besides, the level and functions of some modifications, such as m^6^A, are diverse between cancer cells and infiltrated immune cells.^1,15^ This indicates researchers to separate cancer cells and infiltrated immune cells when analyzing human or animal tumor samples.

### Infectious diseases

Similar to cancers, the pathogenesis and development of infectious diseases are closely related to the immune defense deficiency that involves the deficiency of the immune system itself and immune escape from pathogens.^424–427^ According to existing literature, RNA modifications are critical participators in the progression of infectious diseases by affecting the biology of immune cells.

In recent years, many studies have focused attention on the roles of RNA modifications in viral infection.^53,302,428–430^ On one aspect, RNA modifications, such as m^6^A, m^5^C, ac^4^C, Ψ, and RNA editing, directly act on viral RNAs, thus influencing RNA structure, RNA nuclear export, translation, stability, and replication.^53,430–433^ On the other aspect, RNA modifications can regulate host responses to viral infection by mediating viral RNA sensing and signaling, cytokine responses, as well as immune cell functions, which are emphasis discussion of this text. The roles of RNA modifications in regulating immune cell functions in antiviral infection can also be explicated from two perspectives. The first one is that RNA modifications on viral RNAs repress innate immune signaling pathways. For example, m^6^A-modified HIV-1 and vesicular stomatitis virus RNAs restrain the innate sensing efficacy of MDA5 or RIG-I and thereby impaired IFN-I-mediated innate antiviral immune responses in monocytes and macrophages.^374,379^ The other one is that RNA modifications affect the key factors of antiviral immunity in immune cells, especially in innate immune cells. For example, in monocytes and macrophages, m^6^A modification affects antiviral transcripts including CGAS, IFI16, STING, Mavs, Traf3, Traf6, and FOXO3, as well as signaling pathways including OGDH–itaconate, TBK1–IRF3, and IFN signaling to function in inhibiting viral replication and antiviral innate immunity.^380–382,434^ In NK cells, except for the antitumor activity, YTHDF2 is also essential for the antiviral activity of NK cells by targeting Tardbp.^372^ Especially, as CD4^+^ T cells are the target of HIV infection, RNA modification participates in the viral processes including replication, nuclear export, transcriptional elongation, and viral latency in CD4^+^ T cells via modulating biological processes inside CD4^+^ T cells as described above. After COVID-19 infection, A-to-I editing of endogenous Alu RNAs is decreased in normal human lung cells and in lung biopsies, which may represent the responses of the hosts.^435^ ADAR1 mediated RNA editing on extensive duplex RNA structures can lead to repressed innate immune responses and is profitable for viral replication, which indicates that A-to-I editing prevents autoimmunity while also favoring pathogens (Table 3).^436^

**Table 3:** RNA modifications and immune cells in infectious, inflammatory and autoimmune diseases

Type of immune disease	Involved disease	RNA modifications	Main functions	Ref.
Infectious diseases	HIV-1 and VSV infection	m^6^A	Modified HIV-1 and VSV RNAs restrain the innate sensing efficacy of MDA5 or RIG-I and thereby impaired IFN-I-mediated innate antiviral immune responses in monocytes and macrophages.	^3,14^
	VSV and HSV-1 infection	m^6^A	Inhibits viral replication and antiviral innate immunity via affecting various antiviral transcripts in in monocytes and macrophages.	^380–382,434^
	CMV-1 infection	m^6^A	Essential for the antiviral activity of NK cells by targeting *Tardbp*.	^372^
	COVID-19 infection	A-to-I editing	Edited endogenous Alu RNAs is decreased in normal human lung cells and in lung biopsies, possibly representing the responses of the hosts.	^435^
	Measles virus infection	A-to-I editing	Extensive duplex RNA structure edited by ADAR1 can lead to repressed innate immune responses and is profitable for viral replication.	^436^
	DNA and RNA virus infection	m^6^A, m^5^C, ac^4^C, Ψ, A-to-I editing	Affects RNA structure, RNA nuclear export, translation, stability, and replication.	^53,430–433^
Inflammatory and autoimmune diseases	Hyperhomocysteinemia	m^5^C	NSUN2 upregulates IL-17A expression in an m^5^C-dependent manner in T lymphocytes.	^306^
	Allergic asthma	m^6^A	Participates in the Th1/Th2 imbalance.	^323^
	IBD	m^6^A	Affects immune infiltration and therapeutic response.	^153^
	IBD	m^6^A	Mettl14 deficiency causes impaired induction of naïve T cells into iTreg cells by decreasing RORγt expression, contributing to spontaneous colitis.	^336^
	IBD	A-to-I editing	Impaired A-to-I editing due to *Adar1* in CD4^+^ T cell leads to abnormal thymic T cell maturation and impaired negative selection, resulting in spontaneous colitis.	^307,308^
	Colon and lung Inflammation	m^6^A	IGF2BP2 switches M1 macrophages to M2 activation by stabilizing TSC1 and PPARγ in an m^6^A-dependent manner, leading to inflammatory diseases.	^443^
	Acute lung injury and respiratory distress syndrome	m^6^A	Ablation of METTL14 in myeloid cells exacerbates macrophage responses to acute bacterial infection.	^387^
	Liver fibrosis	m^6^A	Through essentially stimulating pyroptosis and inflammation of macrophages, the signaling cascade METTL3/MALAT1/PTBP1/USP8/TAK1 aggravates liver fibrosis.	^389^
	SLE	m^5^C	m^5^C level and NSUN2 expression are decreased in CD4^+^ T cells, and hypermethylated m^5^C is significantly involved in the immune- and inflammation-related pathways.	^318^
	SLE	ac^4^C	ac^4^C modification in mRNAs of SLE CD4^+^ T cells is highly enriched in CDS regions and involved in the immune and inflammatory signaling of SLE pathogenesis.	^319^
	SLE	A-to-I editing	Up-regulated ADAR1 in SLE T cells is a potential mechanism accounting for the mutations in the RI alpha subunit of type 1 protein kinase A.	^444^
	SLE	A-to-I editing	Involved in generating or elevating the autoantigen load.	^445^
	Autoimmune encephalomyelitis	m^6^A	Ablation of ALKBH5 resulted in increased m^6^A modification on *IFNG* and *CXCL2* mRNA and impaired responses of CD4^+^ T cells, leading to repress autoimmunity.	^315^
	Systemic sclerosis	A-to-I editing	A-to-I editing mediated by ADAR1p150 in PBMCs are closely related to type I IFN responses.	^270^
	Allogeneic transplant	A-to-I editing	Edited RNA can suppress the host antigraft response and promote graft survival through the ADAR1-miR-21-Foxo1-IL-10 axis.	^390,446^

*m*^*6*^*A* *N*^6^-methyladenosine, *m*^*5*^*C* 5-methylcytosine, *ac*^*4*^*C* *N*^4^-acetylcytidine, *ψ* pseudouridine, *A-to-I editing* adenosine-to-inosine RNA editing, *HIV* human immunodeficiency virus, *VSV* vesicular stomatitis virus, *CMV* cytomegalovirus, *COVID-19* Corona Virus Disease 2019, *IBD* inflammatory bowel disease, *SLE* systemic lupus erythematosus

Unfortunately, there is no report on the roles of other RNA modifications other than m^6^A or A-to-I editing in regulating immune cells in infectious diseases. Also, there is few study on the roles of RNA modifications regulating immune cells in infectious diseases induced by other pathogens such as bacteria and fungi. In addition, as we reviewed above, the roles of RNA modifications in antiviral processes are reported only in innate immunity, while its functions in adaptive immunity are ignored and may be of good research interest. Due to the pathogenesis similarity in immune defense deficiency, the research ideas on cancer may provide lessons for infectious diseases in this field.

### Inflammatory and autoimmune diseases

Inflammation and immune responses are critical in opposing harmful stimuli and injury, while their overreaction or being out of control will lead to inflammatory and autoimmune diseases, causing tissue damage and organ dysfunction.^437–439^ Generally speaking, inflammatory diseases and autoimmune diseases are different, but they share some similar pathogenesis,^440–442^ so we discuss them together here. To date, there have some studies verified that RNA modifications may exert biological functions during inflammation and autoimmunity by regulating immune cells.

In hyperhomocysteinemia, NSUN2 upregulates IL-17A expression by inducing *IL-17A* mRNA m^5^C modification in T lymphocytes to mediate chronic inflammation.^306^ In allergic asthma, m^6^A may participate in the Th1/Th2 imbalance.^323^ In inflammatory bowel disease (IBD), m^6^A modification may affect immune infiltration and therapeutic response,^153^ and Mettl14 deficiency can cause impaired induction of naïve T cells into iTreg cells by decreasing RORγt expression, thereby leading to spontaneous colitis.^336^ Deficiency of Adar1 mediated impaired A-to-I editing in CD4^+^ T cell will lead to abnormal thymic T cell maturation and impaired negative selection, thereby resulting in autoimmunity disease such as spontaneous colitis.^307,308^ By regulating various aspects of macrophage biology, including polarization, differentiation, activation, inflammation, and pyroptosis, m^6^A has been found to serve as a mediator in inflammatory diseases including ulcerative colitis, cytokine storm after bacterial infection and liver fibrosis (Table 3).^387,389,443^

In SLE, m^5^C level and NSUN2 expression are decreased in CD4^+^ T cells, and hypermethylated m^5^C is related to the immune- and inflammation-related pathways.^318^ ac^4^C modification in mRNAs of SLE CD4^+^ T cells is highly conserved and enriched in CDS regions and involved in the immune and inflammatory signaling of SLE pathogenesis.^319^ *ADAR1* mRNA was significantly up-regulated in SLE T cells, which may be a potential mechanism accounting for the mutations in the RI alpha subunit of type 1 protein kinase A.^444^ In SLE and some other autoimmune diseases, increased A-to-I RNA editing is involved in generating or elevating the autoantigen load to facilitate autoimmunity progression.^445^ In autoimmune encephalomyelitis, ablation of ALKBH5 resulted in increased m^6^A modification on *IFNG* and *CXCL2* mRNA, as well as impaired responses of CD4^+^ T cells to repress autoimmunity.^315^ In systemic autoimmunity characterized by chronic or acute type I IFN pathway such as systemic sclerosis and other disease contexts, ADAR1p150 isoform mediated A-to-I editing in PBMCs are closely related to type I IFN responses.^270^ ADAR1 can edit putatively immunogenic dsRNA substrates to evade MDA5-mediated dsRNA sensing to suppress inflammatory and autoimmune diseases.^446^ In the allogeneic transplant model, ADAR1 mediated RNA editing can suppress the host antigraft response and promote graft survival through the ADAR1-miR-21-Foxo1-IL-10 axis (Table 3).^390^

Although there are few studies on RNA modification regulating immune cells in inflammatory and autoimmune diseases, as we described above, many studies have confirmed the modulation of RNA modifications on the biology of immune cell closely related to these diseases, which build potential connections between cell biology and human diseases. For example, RNA modifications can influence the biology of DCs, monocytes and macrophages, and mediate inflammatory cytokine secretion,^8,142,365,384,447^ they also probably function in many inflammatory diseases. RNA modifications can affect the activation of T cells and differentiation of Th17 cells, Tfh cells and Treg cells,^142,327,332,337^ which play critical roles in the immune dysregulation of some autoimmune diseases such as SLE and multiple sclerosis,^448,449^ they are likely to participate in the pathogenesis of these diseases. In addition, the research on cancers and infectious diseases listed above may also provide feasible ideas. Due to the contrary roles of immune cell activation and infiltration in cancers and infectious diseases with inflammatory and autoimmune diseases, it is possible that the roles of RNA modifications regulating immune cells are opposite in these diseases.

## Conclusions and perspectives

In this review, we introduced eight RNA modifications including m^6^A, m^5^C, m^1^A, m^7^G, ac^4^C, Ψ, uridylation, and A-to-I editing, and summarized their influence on the biology of immune cells, as well as their roles in immune related diseases by regulating immune cells. The modifications involve various kinds of RNAs, such as mRNAs, noncoding RNAs, tRNAs, rRNAs, or even exogenous RNAs (eg., viral RNAs and synthetic RNAs). These modifications are executed by RNA-modifying enzymes such as writers, erasers and readers, and influence RNA processes including generation, transportation, function, and metabolization. Based on these molecular functions, RNA modifications participate in various biological processes of immune cells, including development, differentiation, activation, migration, and polarization, thus modulating the immune response and participating in the pathogenesis of immune related diseases (Fig. 6 and Table 3).

Currently and a long time in the future, m^6^A will continue to be a research hotspot in this field, especially in the field of anti-tumor immunity, with relatively adequate background knowledge and mature research technology available. In addition, RNA modifications are mostly investigated in T lymphocytes and monocytes/macrophages, which are relatively easy to obtain, and in T cells or monocytes/macrophages related immune processes and diseases. Moreover, due to the global epidemic of COVID-19, RNA modifications that are involved in antiviral immunity may also be of promising research interest.

There remain many questions that should be addressed to fully understand the impact of RNA modifications on immune cell biology. Nevertheless, there is continuous progress in this field and there are presently many meaningful points to study. First, to what extent do RNA modifications regulate immune cell biological processes? Are they the main mediators or auxiliary participants? In other words, how helpful would it to rectify RNA modification abnormalities in the treatment of immune related diseases? This is important for designing therapeutic targets and a critical issue to be solved. Second, some RNA modifications have been proven feasible in the systemic delivery of nanoparticle formulations for regulating both immune cell immunity and inflammation in the laboratory.^309,450,451^ Clinical practice is still the grandest challenge, which will be a valuable research direction. Third, essentially, RNA modifications and their functions are regulated by writers, erasers and readers. However, many studies have failed to elucidate the mechanisms causing the anomalies of these regulators. As RNA modifications can target many downstream RNAs and there are no effective interfering drugs, research on the upstream factors is particularly important. Fourth, further exploration of the role of RNA modifications in various immune cells needs to be done, since the current research is mainly focused on a few modifications (e.g., m^6^A) and immune cells (e.g., T lymphocytes) while overlooking other modifications and immune cells. Immune cells are interconnected and RNA modifications regulate various immune and inflammatory-related factors and signaling pathways. This is a complicated regulatory network that requires us to consummate. Most important, targeting RNA modifications as a treatment in immune related diseases remains in the theoretical stage, and there are no clinical application examples at present. RNA modifications may influence almost all types of RNA, and interfering RNA modifications may cause a wide range of effects. Therefore, gene-specific RNA modification interference is a vital research bottleneck in this area.
